# Supplementary material for: Tracing metal footprints via global renewable power value chains
Source: Nat Commun. 2023 Jun 22;14:3703. doi: 10.1038/s41467-023-39356-x (PMC10287728; doi:10.1038/s41467-023-39356-x)
Supplement: Supplementary file 1 — Supplementary information [file 41467_2023_39356_MOESM1_ESM.pdf]

Supplementary Information

Tracing metal footprints via global renewable power value chains

Rao Fu[1,8], Kun Peng[1,8], Peng Wang[2], Honglin Zhong[1,3], Bin Chen[4], Pengfei Zhang[1], Yiyi Zhang[5], Dongyang Chen[6], Xi Liu[1*], Kuishuang Feng[7*], Jiashuo Li[1,3*]

[1] Institute of Blue and Green Development, Shandong University, Weihai, P. R. China. 264209;

[2] Key Lab of Urban Environment and Health, Institute of Urban Environment, Chinese Academy of Sciences, Xiamen, P. R. China. 361021;

[3] Academy of Plateau Science and Sustainability, Qinghai Normal University, Xining, P. R. China. 810016;

[4] Department of Environmental Science and Engineering, Fudan University, Shanghai, P. R. China. 200082;

[5] Guangxi Key Laboratory of Power System Optimization and Energy Technology, Guangxi University, Nanning, P. R. China. 530004;

[6] Shandong Key Laboratory of Blockchain Finance, Shandong University of Finance and Economics, Jinan, P. R. China. 250014;

[7] Department of Geographical Sciences, University of Maryland, College Park, MD, USA. 20742.

[8] These authors contributed equally

* Correspondence: liuxi@sdu.edu.cn (X.L.), kfeng@umd.edu (K.F.), lijiashuo@sdu.edu.cn (J.L.).

Content

A. Supplementary methods **3**

A.1 Metal use or value-added embodied in global RPVCs 3

A.2 Structural decomposition analysis 5

A.3 Additional data sources 6

B. Supplementary results **6**

B.1 Structural path analysis for per unit of wind power and hydropower generation 6

B.2 The growing metal footprints inequality and its driving forces 7

B.3 Comparison with the results of previous studies 8

C. Supplementary tables **9**

D. Supplementary figures **16**

Supplementary references **29**

A. Supplementary methods

A.1 Metal use or value-added embodied in global RPVCs

It is known that final goods exports can be decomposed into domestic and foreign components using the simple Leontief method[1]. However, the decomposition of intermediate good exports is not so simple[2]. To solve this problem, Wang developed a method to express gross intermediate exports as the demand of different economies according to the destination where they were eventually absorbed[3]. According to input-output model, the gross output of economy r can be decomposed into four parts based on the absorption destination:

$$\mathbf{x}^r = \mathbf{B}^{rr}\mathbf{y}^{rr} + \mathbf{B}^{rr}\mathbf{y}^{rs} + \mathbf{B}^{rs}\mathbf{y}^{sr} + \mathbf{B}^{rs}\mathbf{y}^{ss} \quad (1)$$

Substituting equation (1) into the $\mathbf{A}^{sr}\mathbf{x}^r$ the gross intermediate goods exports in economy s can be expressed as:

$$\mathbf{A}^{sr}\mathbf{x}^r = \mathbf{A}^{sr}\mathbf{B}^{rr}\mathbf{y}^{rr} + \mathbf{A}^{sr}\mathbf{B}^{rr}\mathbf{y}^{rs} + \mathbf{A}^{sr}\mathbf{B}^{rs}\mathbf{y}^{sr} + \mathbf{A}^{sr}\mathbf{B}^{rs}\mathbf{y}^{ss} \quad (2)$$

The basic input-output relationship is as follows:

$$\mathbf{x}^r = \mathbf{A}^{rr}\mathbf{x}^r + \mathbf{y}^{rr} + \mathbf{e}^{rs} \quad (3)$$

After rearranging terms, we have:

$$\mathbf{x}^r = (\mathbf{I} - \mathbf{A}^{rr})^{-1}\mathbf{y}^{rr} + (\mathbf{I} - \mathbf{A}^{rr})^{-1}\mathbf{e}^{rs} \quad (4)$$

Based on where the final products are used, the intermediate metal exports of economy s in equation (2) can be expressed as:

$$\mathbf{A}^{sr}\mathbf{x}^r = \mathbf{A}^{sr}\mathbf{L}^{rr}\mathbf{y}^{rr} + \mathbf{A}^{sr}\mathbf{L}^{rr}\mathbf{e}^{rs} \quad (5)$$

Rearranging terms $\mathbf{A}^{sr}\mathbf{x}^r$, it can be written as:

$$\mathbf{A}^{sr}\mathbf{x}^r = \mathbf{m}^s\mathbf{L}^{ss}\#\mathbf{A}^{sr}\mathbf{x}^r + (\mathbf{m}^s\mathbf{B}^{ss} - \mathbf{m}^s\mathbf{L}^{ss})\#\mathbf{A}^{sr}\mathbf{x}^r + \mathbf{m}^s\mathbf{B}^{rs}\#\mathbf{A}^{sr}\mathbf{x}^r \quad (6)$$

where, "#" represents an elementwise matrix multiplication operation, $\mathbf{m}^s$ *and* $\mathbf{m}^r$ are the direct intensity of metal consumption or the direct value-added coefficient of economy s and r; according to equation (1) in the main text, $\mathbf{m}^s\mathbf{B}^{ss}$ *and* $\mathbf{m}^r\mathbf{B}^{rs}$ represent the domestic and foreign metal consumption or value-added multipliers of economy s (summing up the two terms equal to unity); $\mathbf{m}^s\mathbf{L}^{ss}$ is the local domestic multiplier.

According to Leontief's method, the final product exports of economy s can be expressed as domestic and foreign metal consumption or value-added.

$$\mathbf{y}^{sr} = \mathbf{m}^s\mathbf{B}^{ss}\#\mathbf{y}^{sr} + \mathbf{m}^r\mathbf{B}^{rs}\#\mathbf{y}^{sr} \quad (7)$$

By inserting equations (2) and (5) into equation (6), and combining equations (6)

and (7), we can decompose the exports of economy s as:

$$\mathbf{MEEVC}^{sr} = (\mathbf{m}^s\mathbf{B}^{ss})^T\#\mathbf{y}^{sr} + (\mathbf{m}^s\mathbf{L}^{ss})^T\#(\mathbf{A}^{sr}\mathbf{B}^{rr}\mathbf{y}^{rr}) + (\mathbf{m}^s\mathbf{L}^{ss})^T\#(\mathbf{A}^{sr}\mathbf{B}^{rr}\mathbf{y}^{rs})$$
$$+(\mathbf{m}^s\mathbf{L}^{ss})^T\#(\mathbf{A}^{sr}\mathbf{B}^{rs}\mathbf{y}^{sr}) + (\mathbf{m}^s\mathbf{L}^{ss})^T\#(\mathbf{A}^{sr}\mathbf{B}^{rs}\mathbf{y}^{ss}) + (\mathbf{m}^s\mathbf{B}^{ss} - \mathbf{m}^s\mathbf{L}^{ss})^T\#(\mathbf{A}^{sr}\mathbf{x}^r)$$
$$+\mathbf{m}^r\mathbf{B}^{rs}\#\mathbf{y}^{sr} + (\mathbf{m}^r\mathbf{B}^{rs})^T\#(\mathbf{A}^{sr}\mathbf{L}^{rr}\mathbf{y}^{rr}) + (\mathbf{m}^r\mathbf{B}^{rs})^T\#(\mathbf{A}^{sr}\mathbf{L}^{rr}\mathbf{e}^{rs}) \qquad (8)$$

Extending equation (8) to G economies, the intermediate exports from s to r can be decomposed into eight terms, as in equation (2):

$$\mathbf{A}^{sr}\mathbf{x}^r = \mathbf{A}^{sr}\mathbf{B}^{rr}\mathbf{y}^{rr} + \mathbf{A}^{sr}\sum_{t\neq s,r}^{G}\mathbf{B}^{rt}\mathbf{y}^{tt} + \mathbf{A}^{sr}\mathbf{B}^{rr}\sum_{t\neq s,r}^{G}\mathbf{y}^{rt} + \mathbf{A}^{sr}\sum_{t\neq s,r}^{G}\mathbf{B}^{rt}\sum_{u\neq s,t}^{G}\mathbf{y}^{tu}$$
$$+\mathbf{A}^{sr}\mathbf{B}^{rr}\mathbf{y}^{rs} + \mathbf{A}^{sr}\sum_{t\neq s,r}^{G}\mathbf{B}^{rt}\mathbf{y}^{ts} + \mathbf{A}^{sr}\mathbf{B}^{rs}\mathbf{y}^{ss} + \mathbf{A}^{sr}\sum_{t\neq s}^{G}\mathbf{B}^{rs}\mathbf{y}^{st} \qquad (9)$$

For equation (7), the final product exports of economy s for G economies can be expressed as:

$$\mathbf{y}^{sr} = \mathbf{m}^s\mathbf{B}^{ss}\#\mathbf{y}^{sr} + \mathbf{m}^r\mathbf{B}^{rs}\#\mathbf{y}^{sr} + \sum_{t\neq s,r}^{G}\mathbf{m}^t\mathbf{B}^{ts}\#\mathbf{y}^{sr} \qquad (10)$$

Combining equations (9) and (10), the total bilateral trade of metal use or value-added (export from s to r as example) can be written as equation (5) in the main text.

As illustrated above and in the main text, we combined a multi-regional input-output model (MRIO) with a value chain decomposition model, which enables us to quantify the metal use or value added associated with renewable power sector within each value chain route. Specifically, following Koopman et al. (2014)[4], and Wang et al. (2013)[3], the bilateral trade flows can be decomposed into five parts according to the final consumption destination and product types (intermediate goods with box shaded with grey color and final goods with box shaded with blue color in Supplementary Figure 11). Supplementary Figure 11 comprehensively shows how an economy's gross exports generate both domestic and foreign metal use or economic gains to satisfy renewable power demand through five global value chain routes.

The five paths can be classified into simple or complex value chains, for which, the traded products cross border only once (paths 1 and 2), or at least twice (paths 3, 4 and 5). As an example of the simple value chain, the path 2 in Supplementary Figure 11, indicates that economy s' domestic intermediate goods are consumed by direct

importer r. An example in the wind power sector is that the Australian's (economy s) iron ores are exported to China (economy r), being manufactured and eventually used in wind farms for electricity generation and consumption in China. For the complex value chains, for example, the path 5 describes that economy s uses the intermediate products from the third economy t to produce the intermediate exports, which is further processed and re-exported to economy r for final consumption. An example for path 5 is that the iron ores are exported from Australia (third economy t) to China (economy s), which is processed into steel in China and then exported to the United States (economy r). The steel is used as inputs for wind tower manufacturing, and finally used for wind power generation and consumption in the United States.

A.2 Structural decomposition analysis

In the structural decomposition analysis model, the changes in metals embodied in exports (MEE) and metals embodied in imports (MEI) were disentangled over time, and the contribution of a certain factor was quantified[5]. Here, we further distinguish the drivers between the local and abroad economies to quantify their differentiated contributions to the changes in MEE and MEI. Specifically, the direct metal intensity (**m**), production technology (**H**) ($\mathbf{h}^r = \sum_{s=1}^{N} \mathbf{A}^{sr}$), trade structure of intermediate inputs (**T**) ($t_{ij}^{sr} = a_{ij}^{sr}/h_{ij}^{r}$), demand (**y**) ($\mathbf{y}^{\mathrm{r}} = \sum_{s=1}^{N} \mathbf{y}^{sr}$), and trade structure (**D**) ($d_j^{sr} = y_j^{sr}/y_j^{r}$) are included.

We define the case of economy r (r = 1, …, N) as follows:

$$\begin{gathered}\mathbf{m} = \mathbf{m}^{(r)} + \mathbf{m}^{(-r)} \\ \mathbf{T} = \mathbf{T}^{(r)} + \mathbf{T}^{(-r)} \\ \mathbf{H} = \mathbf{H}^{(r)} + \mathbf{H}^{(-r)} \\ \mathbf{D} = \mathbf{D}^{(r)} + \mathbf{D}^{(-r)} \\ \mathbf{y} = \mathbf{y}^{(r)} + \mathbf{y}^{(-r)}\end{gathered} \tag{11}$$

Specifically, the vector $\mathbf{m}^{(r)}$ only contains the elements of the metal coefficients at home and the other elements are zero, $(\mathbf{m}^{(r)})' = \left[0' \ldots 0'(\mathbf{m}^{(r)})' \ldots 0'\right]'$. The vector $\mathbf{m}^{(-r)}$ contains the elements of the metal coefficients abroad and that of economy r

becomes zero, $(\mathbf{m}^{(-r)})' = \left[(\mathbf{m}^{(1)})' \dots (\mathbf{m}^{(r-1)})' \, 0' \, (\mathbf{m}^{(r+1)})' \dots (\mathbf{m}^{(N)})'\right]'$. A similar structure was used to decompose other factors.

A.3 Additional data sources

For a more reliable accounting, the mineral ores extraction data of economies (e.g., Austria and Indonesia) in 2015 included in the environmental satellite accounts in EXIOBASE, were cross-checked and updated based on the latest database, national minerals yearbook[6,7] published by the U.S. Geological Survey and World Mineral Production 2014—2018[8] developed by the British Geological Survey. The technical coefficient matrix for the production of electricity by biomass and waste sector in Austria was also adjusted following that of Germany to avoid overestimation. For calculating the metal intensity (total supply chain metal use per unit of renewable electricity generation, kg/MWh) in each economy in Supplementary Table 1, the total supply chain metal use of gross renewable electricity production of each economy was obtained based on author's calculation. The gross renewable electricity generation and installed capacity data for the corresponding year were derived from the International Renewable Energy Agency (IRENA)[9]. Furthermore, the GDP and total population data for the corresponding year were derived from the World Bank[10], and the GDP in US dollar is converted to EUR based on the exchange rate in corresponding year.

B. Supplementary results

B.1 Structural path analysis for per unit of wind power and hydropower generation

We employ the structural path analysis (SPA) to analyze the production network through inter-sector flows, which enables us to investigate the crucial paths for metal ores use driven by renewable power demand along the supply chain[11,12]. Accordingly, Supplementary Figure 4 depicts the direct and indirect economic activities induced by one unit (i.e., EUR €1) of wind power or hydropower generation from different production layers, which provides information for how the metal ores are used directly or indirectly. For hydropower, the ratio of indirect metal use to the total metal use per unit of hydropower generation was more than 60%, in which the iron ores and copper

ores accounted for approximately half in China and RoW America (Latin American economies, excluding Brazil), respectively. Specifically, the iron ores consumption was mainly induced by the iron ore-intensive electrical machinery and equipment and fabricated metal products manufacturing sectors in China. Differently, the copper ores consumption was mostly driven by the copper ore-intensive copper mining and construction sectors in RoW America (Latin American economies, excluding Brazil).

B.2 The growing metal footprints inequality and its driving forces

Figure 4 and Supplementary Figures 11 and 12 illustrate the contributions of trade structure and production technology to the changes in metals embodied in export (MEE) and import (MEI) for economies from 2005 to 2015. The changes in production technology and trade structure contributed to a moderate growth of metal inequality. Production technology shifts ($\mathbf{H}^{(r)}$, $\mathbf{H}^{(-r)}$) caused the MEI increase in rich economies (major importers) by 41%–200%, 13%–145%, and MEE growth in most developing economies (major exporters) by 4%–76%, 14%–99%, respectively. One possible reason is that more intermediate inputs than value-added are required per unit of renewable power production globally from 2005 to 2015. The other could be that production technology shifted to a more metal-intensive pattern driven by the developed economies. For instance, the offshore wind power capacity in EU-15 increased by 15 times in 2005—2015, which required around 2 times of the metal inputs for onshore wind power. In contrast, production technology shifts also contributed to the decrease of both MEI in developed economies (e.g., USA and Austria) and MEE in developing economies (e.g., Brazil and Russia). Notably, the MEI increase in Japan was mostly attributed to the trade structure of intermediate goods at home ($\mathbf{T}^{(r)}$), with a contribution of 526%, much higher than the global average, 25%. It also generated a moderate MEE increase in some developing economies (major exporters) by 8%–279%. Japan tended to buying more intermediate goods where they were produced in a high metal-intensive fashion (e.g., from RoW Asia or China) from 2005 to 2015 and induce the positive effect, resulting deeper inequality between Japan and others.

B.3 Comparison with the results of previous studies

Although the calculated metal intensity (total supply chain metal use per unit of renewable electricity generation, kg/MWh) in our study are comparable to that from Hertwich et al. (2015)[13], for some metals such as copper, the intensities are 2–3 times bigger than Hertwich et al. (2015)'s (Supplementary Figure 12). The differences can be explained by several factors: (a) Differences in the scope of metal. In Hertwich et al. (2015), the metal can be understood as the metal content of the ores extracted and utilized for primary production and of the waste streams utilized for secondary production. In comparison, the extraction of gross ore (run of mine) calculated via the ore grades (or conversion factors) reported for each metal in each economy was used in this study. (b) Regional aggregation. Hertwich et al. (2015) constructed a nine-region multi-regional input-output (MRIO) model based on the EXIOBASE MRIO tables. Differently, the detailed EXIOBASE MRIO tables (forty-nine-economy) was employed in this study. The detailed estimation of MFs of each economy can help us gauge the regional heterogeneity and mitigate the impacts of regional aggregation error, resulting in slight differences in the results.

C. **Supplementary tables**

Supplementary Table 1. The GDP per capita (EUR per person) and metal intensity (for renewable power sector, kg/MWh) in 2015 for economies in EXIOBASE.

No.	Abbre-viation	Full name	GDP per capita	Metal intensity	Economy categories
1	AUT	Austria	39296	4.41	Europe
2	BEL	Belgium	36462	0.60	Europe
3	BGR	Bulgaria	6276	0.32	Europe
4	CYP	Cyprus	20822	0.42	Middle East
5	CZE	Czech Republic	15860	0.96	Europe
6	DEU	Germany	36547	0.46	Europe
7	DNK	Denmark	47370	0.20	Europe
8	EST	Estonia	15473	1.92	Europe
9	ESP	Spain	22889	0.85	Europe
10	FIN	Finland	38057	0.38	Europe
11	FRA	France	32590	0.97	Europe
12	GRC	Greece	16056	0.04	Europe
13	HRV	Croatia	10480	0.87	Europe
14	HUN	Hungary	11303	1.78	Europe
15	IRL	Ireland	55138	0.38	Europe
16	ITA	Italy	26890	1.13	Europe
17	LTU	Lithuania	12683	1.07	Europe
18	LUX	Luxembourg	90174	18.80	Europe
19	LVA	Latvia	12253	0.55	Europe
20	MLT	Malta	22168	11.98	Europe
21	NLD	Netherlands	40183	2.31	Europe
22	POL	Poland	11189	1.37	Europe
23	PRT	Portugal	17116	1.44	Europe
24	ROM	Romania	7978	0.33	Europe
25	SWE	Sweden	45850	0.32	Europe
26	SVN	Slovenia	18574	0.80	Europe
27	SVK	Slovakia	14509	2.67	Europe
28	GBR	United Kingdom	40062	0.52	Europe
29	USA	United States	50580	0.09	North America
30	JPN	Japan	31097	0.50	Other Asia
31	CHN	China	7176	2.20	China
32	CAN	Canada	38779	0.11	North America
33	KOR	South Korea	25557	0.50	Other Asia
34	BRA	Brazil	7840	0.02	Latin America
35	IND	India	1428	0.20	India
36	MEX	Mexico	8554	0.35	North America
37	RUS	Russia	8284	0.08	Russia
38	AUS	Australia	50484	0.13	Australia
39	CHE	Switzerland	75408	1.19	Europe
40	TUR	Turkey	9790	0.28	Middle East
41	TWN	Taiwan	41767	1.38	China
42	NOR	Norway	66139	0.18	Europe
43	IDN	Indonesia	2964	0.04	Other Asia
44	ZAF	South Africa	5101	0.08	Africa
45	WWA	RoW Asia and Pacific	5795	0.55	Other Asia
46	WWL	RoW America	7677	0.59	Latin America
47	WWE	RoW Europe	6712	0.10	Europe
48	WWF	RoW Africa	1726	1.64	Africa
49	WWM	RoW Middle East	9178	3.00	Middle East

Note: The average metal intensity of seven renewable power sectors differs across economies due to the varieties in their renewable power types, supply-chain production processes and so on[13].

Supplementary Table 2. GNI per capita for each economy and the economy classifications.

No.	Economy name	GNI per capita	Economy categories	Income group
1	Austria	47480	Developed economies	High Income
2	Belgium	45570	Developed economies	High Income
3	Bulgaria	7430	Developing economies	Upper middle income
4	Cyprus	26010	Developed economies	High Income
5	Czech Republic	18370	Developed economies	High Income
6	Germany	45780	Developed economies	High Income
7	Denmark	60510	Developed economies	High Income
8	Estonia	18570	Developed economies	High Income
9	Spain	28450	Developed economies	High Income
10	Finland	47180	Developed economies	High Income
11	France	41130	Developed economies	High Income
12	Greece	20190	Developed economies	High Income
13	Croatia	13270	Developed economies	High Income
14	Hungary	13230	Developed economies	High Income
15	Ireland	50390	Developed economies	High Income
16	Italy	33000	Developed economies	High Income
17	Lithuania	15190	Developed economies	High Income
18	Luxembourg	73530	Developed economies	High Income
19	Latvia	15110	Developed economies	High Income
20	Malta	25230	Developed economies	High Income
21	Netherlands	49850	Developed economies	High Income
22	Poland	13310	Developed economies	High Income
23	Portugal	20460	Developed economies	High Income
24	Romania	9600	Developing economies	Upper middle income
25	Sweden	58420	Developed economies	High Income
26	Slovenia	22270	Developed economies	High Income
27	Slovakia	17700	Developed economies	High Income
28	United Kingdom	44730	Developed economies	High Income
29	United States	56620	Developed economies	High Income
30	Japan	39380	Developed economies	High Income
31	China	7890	Developing economies	Upper middle income
32	Canada	47590	Developed economies	High Income
33	South Korea	28720	Developed economies	High Income
34	Brazil	10170	Developing economies	Upper middle income
35	India	1600	Developing economies	Lower middle income
36	Mexico	10160	Developing economies	Upper middle income
37	Russia	11780	Developing economies	Upper middle income
38	Australia	60490	Developed economies	High Income
39	Switzerland	87960	Developed economies	High Income
40	Turkey	12030	Developing economies	Upper middle income
41	Taiwan	23108	Developed economies	High Income
42	Norway	92910	Developed economies	High Income
43	Indonesia	3430	Developing economies	Lower middle income
44	South Africa	6610	Developing economies	Upper middle income
45	RoW Asia and Pacific	3357	Developing economies	Lower middle income
46	RoW America	6994	Developing economies	Upper middle income
47	RoW Europe	3546	Developing economies	Lower middle income
48	RoW Africa	1764	Developing economies	Lower middle income
49	RoW Middle East	10348	Developing economies	Upper middle income

Note: Using World Bank's classification based on GNI (Gross National Income, US$) per capita in 2015[10], we classify 49 EXIOBASE economies into 34 developed economies (i.e., high income economies > 12475 US$) and 15 developing economies (i.e., upper middle income economies, 4036-12475 US$, lower middle income economies, 1026-4035 US$, and low income economies <= 1025 US$)[14].

Supplementary Table 3. The remaining international flows of metals embodied in renewable power value chains among ten groups of economies in 2005, unit: kt.

No.	Origin–destination	Embodied metal flows	No.	Origin–destination	Embodied metal flows
1	Europe–Middle East	14.04	40	Other Asia–Middle East	4.67
2	Europe–North America	6.12	41	Other Asia–Africa	2.96
3	Europe–Other Asia	2.87	42	Other Asia–Latin America	1.63
4	Europe–Africa	1.37	43	Other Asia–Australia	0.89
5	Europe–Latin America	0.58	44	Other Asia–India	7.33
6	Europe–Australia	0.19	45	Other Asia–Russia	3.36
7	Europe–China	5.31	46	Latin America–Other Asia	19.60
8	Europe–India	1.19	47	Latin America–Africa	5.28
9	Europe–Russia	0.92	48	Latin America–Australia	0.74
10	China–Middle East	3.73	49	Latin America–India	6.61
11	China–North America	9.59	50	Latin America–Russia	2.70
12	China–Africa	2.15	51	North America–Europe	21.13
13	China–Latin America	1.12	52	North America–Middle East	2.15
14	China–Australia	0.18	53	North America–Other Asia	3.81
15	China–India	2.74	54	North America–Africa	0.64
16	China–Russia	0.50	55	North America–Latin America	1.21
17	Australia–Middle East	2.52	56	North America–Australia	0.08
18	Australia–North America	7.55	57	North America–India	12.20
19	Australia–Africa	2.05	58	North America–Russia	0.95
20	Australia–Latin America	1.10	59	Africa–Middle East	1.76
21	Australia–India	4.91	60	Africa–North America	4.31
22	Australia–Russia	0.77	61	Africa–Other Asia	4.17
23	Russia–Middle East	4.42	62	Africa–Latin America	0.51
24	Russia–North America	4.41	63	Africa–Australia	0.21
25	Russia–Other Asia	3.77	64	Africa–China	9.97
26	Russia–Africa	1.06	65	Africa–India	1.36
27	Russia–Latin America	0.49	66	Africa–Russia	0.54
28	Russia–Australia	0.11	67	Middle East–Europe	8.96
29	Russia–China	16.92	68	Middle East–North America	2.25
30	Russia–India	1.34	69	Middle East–Other Asia	1.64
31	India–Europe	12.63	70	Middle East–Africa	1.01
32	India–Middle East	1.51	71	Middle East–Latin America	0.16
33	India–North America	2.29	72	Middle East–Australia	0.06
34	India–Other Asia	4.62	73	Middle East–India	2.76
35	India–Africa	1.26	74	Middle East–Russia	1.38
36	India–Latin America	0.33			
37	India–Australia	0.06			
38	India–China	24.57			
39	India–Russia	0.15			

Supplementary Table 4. The remaining international flows of metals embodied in renewable power value chains among ten groups of economies in 2015, units: kt.

No.	Origin–destination	Embodied metal flows	No.	Origin–destination	Embodied metal flows
1	Europe–Middle East	16.45	39	Other Asia–Middle East	5.90
2	Europe–North America	4.93	40	Other Asia–North America	5.59
3	Europe–Other Asia	6.00	41	Other Asia–Africa	4.50
4	Europe–Africa	2.95	42	Other Asia–Latin America	26.28
5	Europe–Latin America	0.94	43	Other Asia–Australia	0.67
6	Europe–Australia	0.21	44	Other Asia–India	2.95
7	Europe–China	35.52	45	Other Asia–Russia	4.12
8	Europe–India	0.85	46	Latin America–Middle East	28.22
9	Europe–Russia	0.44	47	Latin America–Other Asia	2.06
10	China–Middle East	7.54	48	Latin America–Africa	8.48
11	China–North America	8.43	49	Latin America–Australia	0.47
12	China–Africa	11.43	50	Latin America–India	4.20
13	China–Latin America	5.82	51	Latin America–Russia	0.74
14	China–Australia	0.52	52	North America–Middle East	2.17
15	China–India	4.39	53	North America–Other Asia	7.29
16	China–Russia	0.35	54	North America–Africa	2.34
17	Australia–Middle East	5.60	55	North America–Latin America	3.74
18	Australia–North America	5.60	56	North America–Australia	0.14
19	Australia–Africa	6.70	57	North America–India	1.01
20	Australia–Latin America	3.53	58	North America–Russia	0.21
21	Australia–India	4.10	59	Africa–Middle East	4.31
22	Australia–Russia	0.37	60	Africa–North America	6.18
23	Russia–Middle East	2.89	61	Africa–Other Asia	9.48
24	Russia–North America	2.02	62	Africa–Latin America	1.27
25	Russia–Other Asia	3.15	63	Africa–Australia	0.23
26	Russia–Africa	1.17	64	Africa–India	2.07
27	Russia–Latin America	0.46	65	Africa–Russia	0.28
28	Russia–Australia	0.06	66	Middle East–Europe	17.97
29	Russia–India	0.44	67	Middle East–North America	1.59
30	India–Europe	13.20	68	Middle East–Other Asia	3.52
31	India–Middle East	2.60	69	Middle East–Africa	2.13
32	India–North America	0.82	70	Middle East–Latin America	0.45
33	India–Other Asia	2.81	71	Middle East–Australia	0.13
34	India–Africa	1.94	72	Middle East–India	1.04
35	India–Latin America	0.52	73	Middle East–Russia	0.10
36	India–Australia	0.04			
37	India–China	15.34			
38	India–Russia	0.04			

Supplementary Table 5. The sector classification in EXIOBASE and renewable power sectors (99–100, and 102–106).

No.	Sectors	No.	Sectors
1	Cultivation of paddy rice	83	Re-processing of secondary other non-ferrous metals into new other non-ferrous metals
2	Cultivation of wheat	84	Casting of metals
3	Cultivation of cereal grains nec	85	Manufacture of fabricated metal products, except machinery and equipment
4	Cultivation of vegetables, fruit, nuts	86	Manufacture of machinery and equipment nec
5	Cultivation of oil seeds	87	Manufacture of office machinery and computers
6	Cultivation of sugar cane, sugar beet	88	Manufacture of electrical machinery and apparatus nec
7	Cultivation of plant-based fibers	89	Manufacture of radio, television and communication equipment and apparatus
8	Cultivation of crops nec	90	Manufacture of medical, precision and optical instruments, watches and clocks
9	Cattle farming	91	Manufacture of motor vehicles, trailers and semi-trailers
10	Pigs farming	92	Manufacture of other transport equipment
11	Poultry farming	93	Manufacture of furniture; manufacturing nec
12	Meat animals nec	94	Recycling of waste and scrap
13	Animal products nec	95	Recycling of bottles by direct reuse
14	Raw milk	96	Production of electricity by coal
15	Wool, silk-worm cocoons	97	Production of electricity by gas
16	Manure treatment (conventional), storage and land application	98	Production of electricity by nuclear
17	Manure treatment (biogas), storage and land application	99	Production of electricity by hydro
18	Forestry, logging and related service activities	100	Production of electricity by wind
19	Fishing, operating of fish hatcheries and fish farms; service activities incidental to fishing	101	Production of electricity by petroleum and other oil derivatives
20	Mining of coal and lignite; extraction of peat	102	Production of electricity by biomass and waste
21	Extraction of crude petroleum and services related to crude oil extraction, excluding surveying	103	Production of electricity by solar photovoltaic
22	Extraction of natural gas and services related to natural gas extraction, excluding surveying	104	Production of electricity by solar thermal
23	Extraction, liquefaction, and regasification of other petroleum and gaseous materials	105	Production of electricity by tide, wave, ocean
24	Mining of uranium and thorium ores	106	Production of electricity by Geothermal
25	Mining of iron ores	107	Production of electricity nec
26	Mining of copper ores and concentrates	108	Transmission of electricity
27	Mining of nickel ores and concentrates	109	Distribution and trade of electricity
28	Mining of aluminium ores and concentrates	110	Manufacture of gas; distribution of gaseous fuels through mains

29	Mining of precious metal ores and concentrates	111	Steam and hot water supply
30	Mining of lead, zinc and tin ores and concentrates	112	Collection, purification and distribution of water
31	Mining of other non-ferrous metal ores and concentrates	113	Construction
32	Quarrying of stone	114	Re-processing of secondary construction material into aggregates
33	Quarrying of sand and clay	115	Sale, maintenance, repair of motor vehicles, motor vehicles parts, motorcycles, motor cycles parts and accessories
34	Mining of chemical and fertilizer minerals, production of salt, other mining and quarrying nec	116	Retail sale of automotive fuel
35	Processing of meat cattle	117	Wholesale trade and commission trade, except of motor vehicles and motorcycles
36	Processing of meat pigs	118	Retail trade, except of motor vehicles and motorcycles; repair of personal and household goods
37	Processing of meat poultry	119	Hotels and restaurants
38	Production of meat products nec	120	Transport via railways
39	Processing vegetable oils and fats	121	Other land transport
40	Processing of dairy products	122	Transport via pipelines
41	Processed rice	123	Sea and coastal water transport
42	Sugar refining	124	Inland water transport
43	Processing of Food products nec	125	Air transport
44	Manufacture of beverages	126	Supporting and auxiliary transport activities; activities of travel agencies
45	Manufacture of fish products	127	Post and telecommunications
46	Manufacture of tobacco products	128	Financial intermediation, except insurance and pension funding
47	Manufacture of textiles	129	Insurance and pension funding, except compulsory social security
48	Manufacture of wearing apparel; dressing and dyeing of fur	130	Activities auxiliary to financial intermediation
49	Tanning and dressing of leather; manufacture of luggage, handbags, saddlery, harness and footwear	131	Real estate activities
50	Manufacture of wood and of products of wood and cork, except furniture; manufacture of articles of straw and plaiting materials	132	Renting of machinery and equipment without operator and of personal and household goods
51	Re-processing of secondary wood material into new wood material	133	Computer and related activities
52	Pulp	134	Research and development
53	Re-processing of secondary paper into new pulp	135	Other business activities
54	Paper	136	Public administration and defence; compulsory social security
55	Publishing, printing and reproduction of recorded media	137	Education
56	Manufacture of coke oven products	138	Health and social work
57	Petroleum Refinery	139	Incineration of waste: Food
58	Processing of nuclear fuel	140	Incineration of waste: Paper
59	Plastics, basic	141	Incineration of waste: Plastic

60	Re-processing of secondary plastic into new plastic	142	Incineration of waste: Metals and Inert materials
61	N-fertiliser	143	Incineration of waste: Textiles
62	P- and other fertiliser	144	Incineration of waste: Wood
63	Chemicals nec	145	Incineration of waste: Oil/Hazardous waste
64	Manufacture of rubber and plastic products	146	Biogasification of food waste, incl. land application
65	Manufacture of glass and glass products	147	Biogasification of paper, incl. land application
66	Re-processing of secondary glass into new glass	148	Biogasification of sewage slugde, incl. land application
67	Manufacture of ceramic goods	149	Composting of food waste, incl. land application
68	Manufacture of bricks, tiles and construction products, in baked clay	150	Composting of paper and wood, incl. land application
69	Manufacture of cement, lime and plaster	151	Waste water treatment, food
70	Re-processing of ash into clinker	152	Waste water treatment, other
71	Manufacture of other non-metallic mineral products nec	153	Landfill of waste: Food
72	Manufacture of basic iron and steel and of ferro-alloys and first products thereof	154	Landfill of waste: Paper
73	Re-processing of secondary steel into new steel	155	Landfill of waste: Plastic
74	Precious metals production	156	Landfill of waste: Inert/metal/hazardous
75	Re-processing of secondary precious metals into new precious metals	157	Landfill of waste: Textiles
76	Aluminium production	158	Landfill of waste: Wood
77	Re-processing of secondary aluminium into new aluminium	159	Activities of membership organisation nec
78	Lead, zinc and tin production	160	Recreational, cultural and sporting activities
79	Re-processing of secondary lead into new lead	161	Other service activities
80	Copper production	162	Private households with employed persons
81	Re-processing of secondary copper into new copper	163	Extra-territorial organizations and bodies
82	Other non-ferrous metal production		

Note: For illustrative purpose, some sectors are combined into one single sector and shown in Supplementary Figure 4. Specifically, 20–23, 89–95 are combined into Mining of fuel resources, 24, 27–34 are combined into Mining of other minerals, 25, 87 are combined into Mining of iron ores, 26, 86 are combined into Mining of copper ores and concentrates, 47–71 are combined into Non-metallic mineral products, 72–73 are combined into Basic iron and steel and ferro-alloys and first products, 74–79, 82–84 are combined into Other non-ferrous metal production, 80–81 are combined into Copper production, 107–112 are combined into Transmission of electricity & Manufacture of gas, 113–114 are combined into Construction, 120–126 are combined into Transport sectors.

D. Supplementary figures

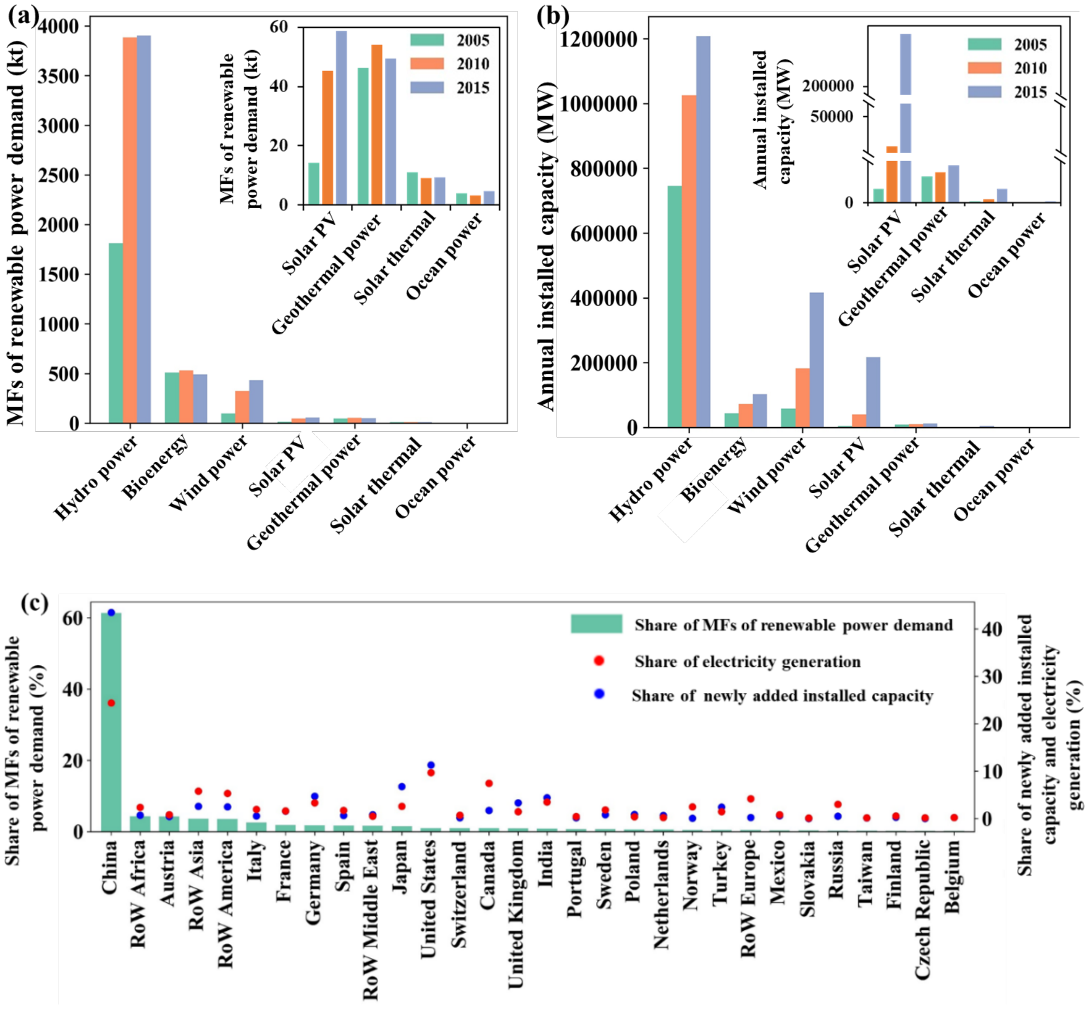

Supplementary Figure 1. The annual metal footprints (MFs) and installed capacity of renewable power sectors in 2005, 2010 and 2015; the shares of MFs, newly added installed capacity, and electricity generation in global total in 2015. (a) Annual MFs, (b) Annual installed capacity. The solar photovoltaic (PV), geothermal power, solar thermal and ocean power are placed in the insets due to the lower value compared with other sectors. (c) The shares of MFs, newly added installed capacity, and electricity generation in global total for top 30 economies, with rankings based on the MFs of all renewable power demand (98% of the global total MFs).

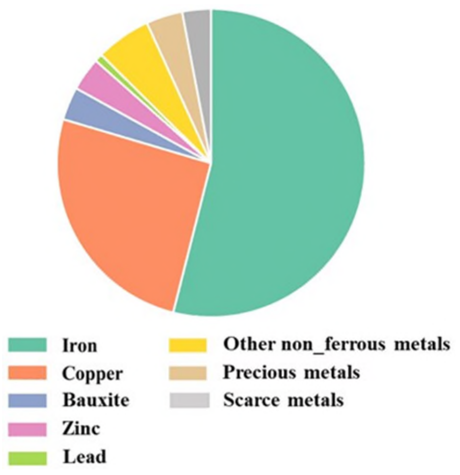

Supplementary Figure 2. Global total metal footprints of renewable power demand by metal types in 2015.

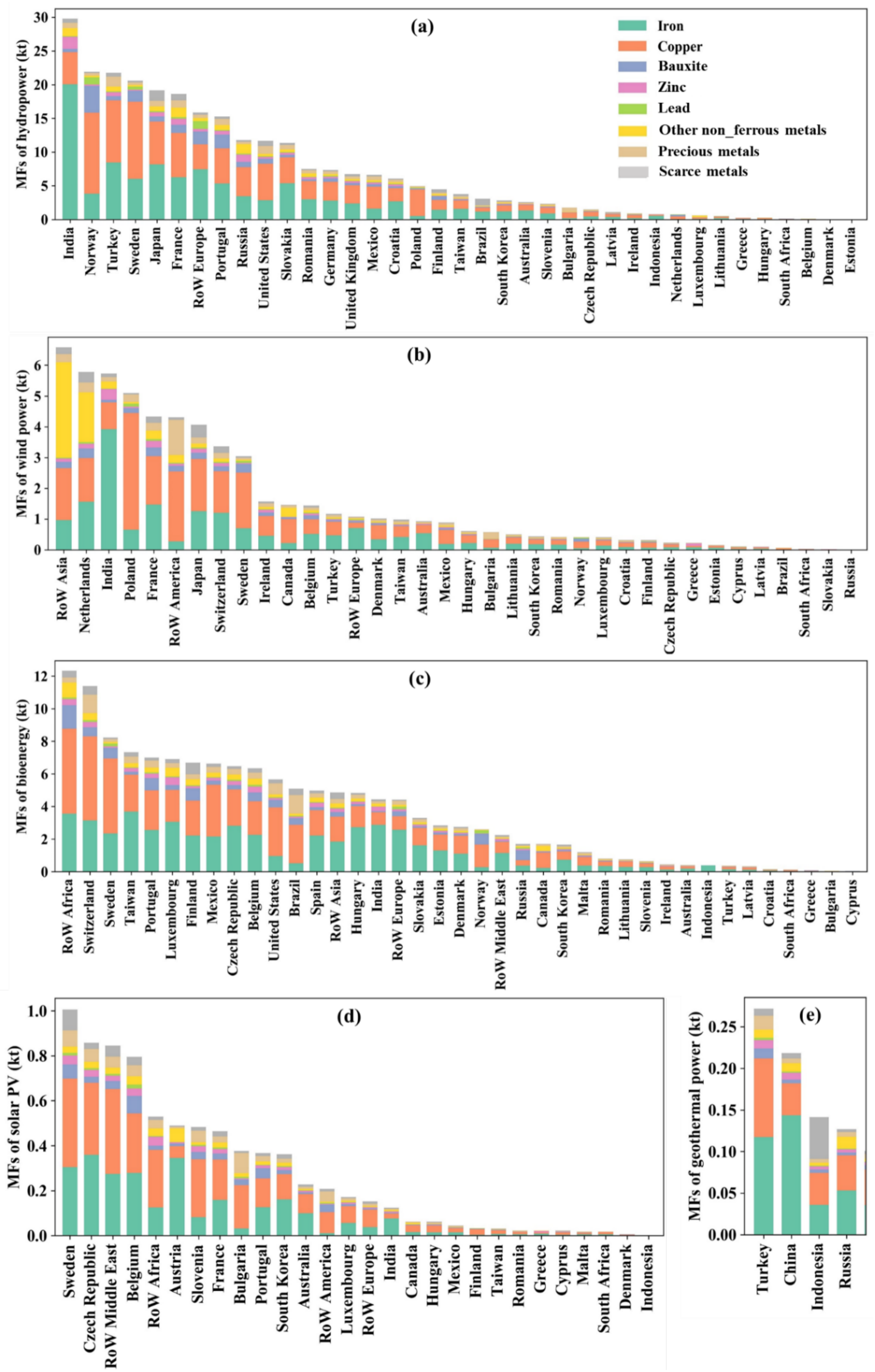

Supplementary Figure 3. Metal footprints (MFs) of the renewable power demand in remaining economies by metal types in 2015. The bottom 39 economies in MFs of (a) hydropower, (b) wind power, (c) bioenergy, (d) solar photovoltaic (PV) and (e) geothermal power. All economies are shown unless the MFs is 0.

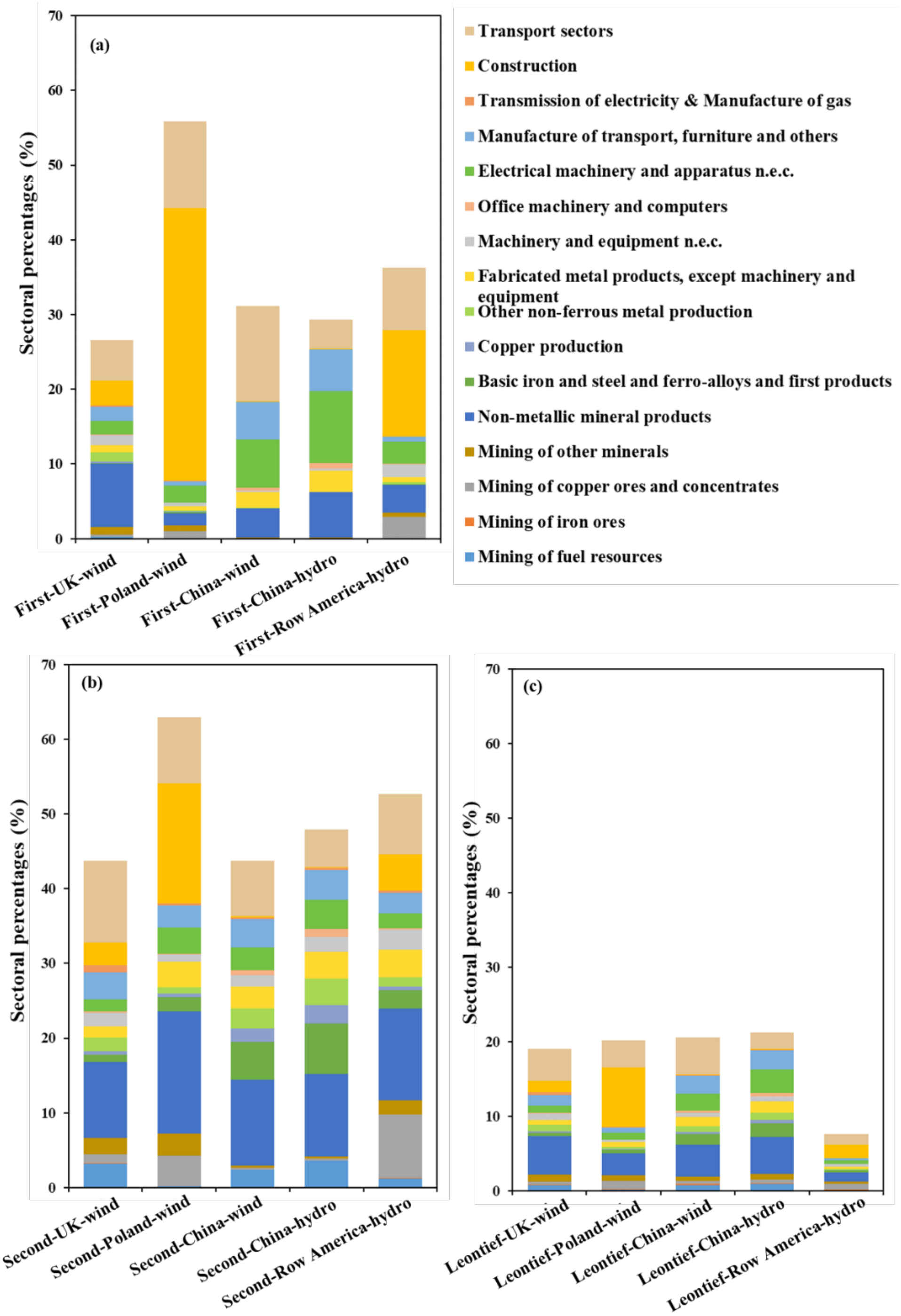

Supplementary Figure 4. The sectoral percentages in economy activities induced by one unit of wind power or hydropower generation for selected economies in 2015. (a) The direct sector inputs for wind power or hydropower generation from the first production layer. (b) The indirect sector inputs for wind power or hydropower generation from the second production layer. (c) The total direct and indirect sector inputs for wind power or hydropower generation, estimated via the Leontief inverse matrix. For illustrative purpose, only those sectors (see Supplementary Table 5) closely related to the metal ores consumption are represented, such as mining, manufacturing, construction, and transportation sectors.

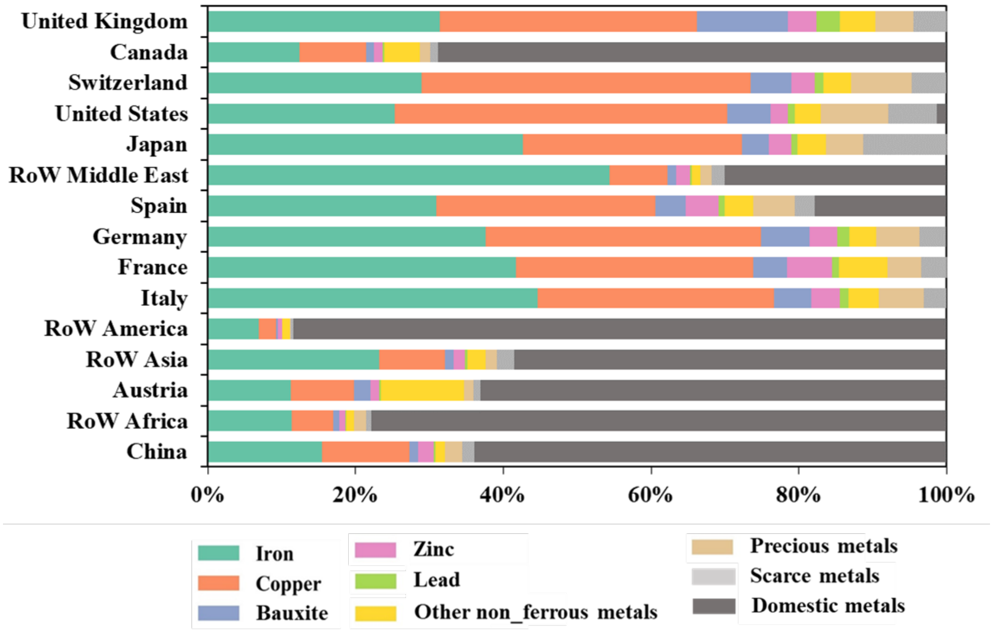

Supplementary Figure 5. Metal types and sources for the top 15 economies in terms of metal footprints of renewable power demand in 2015. The black bar represents the domestic total metal ores and the rest bar represents the metal ores outsourced to other economies. Precious metals include silver and platinum-group metals; Scarce metals include nickel and tin.

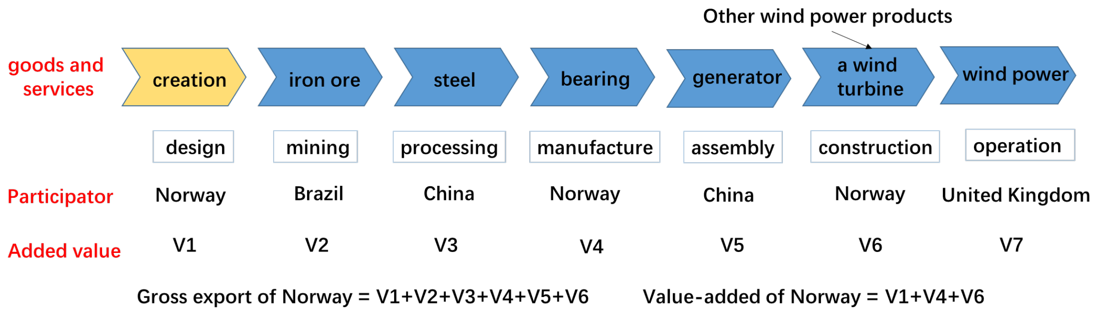

Supplementary Figure 6. An example of value-added distribution of Norway's gross exports of goods and services to satisfy the United Kingdom's wind power demand.

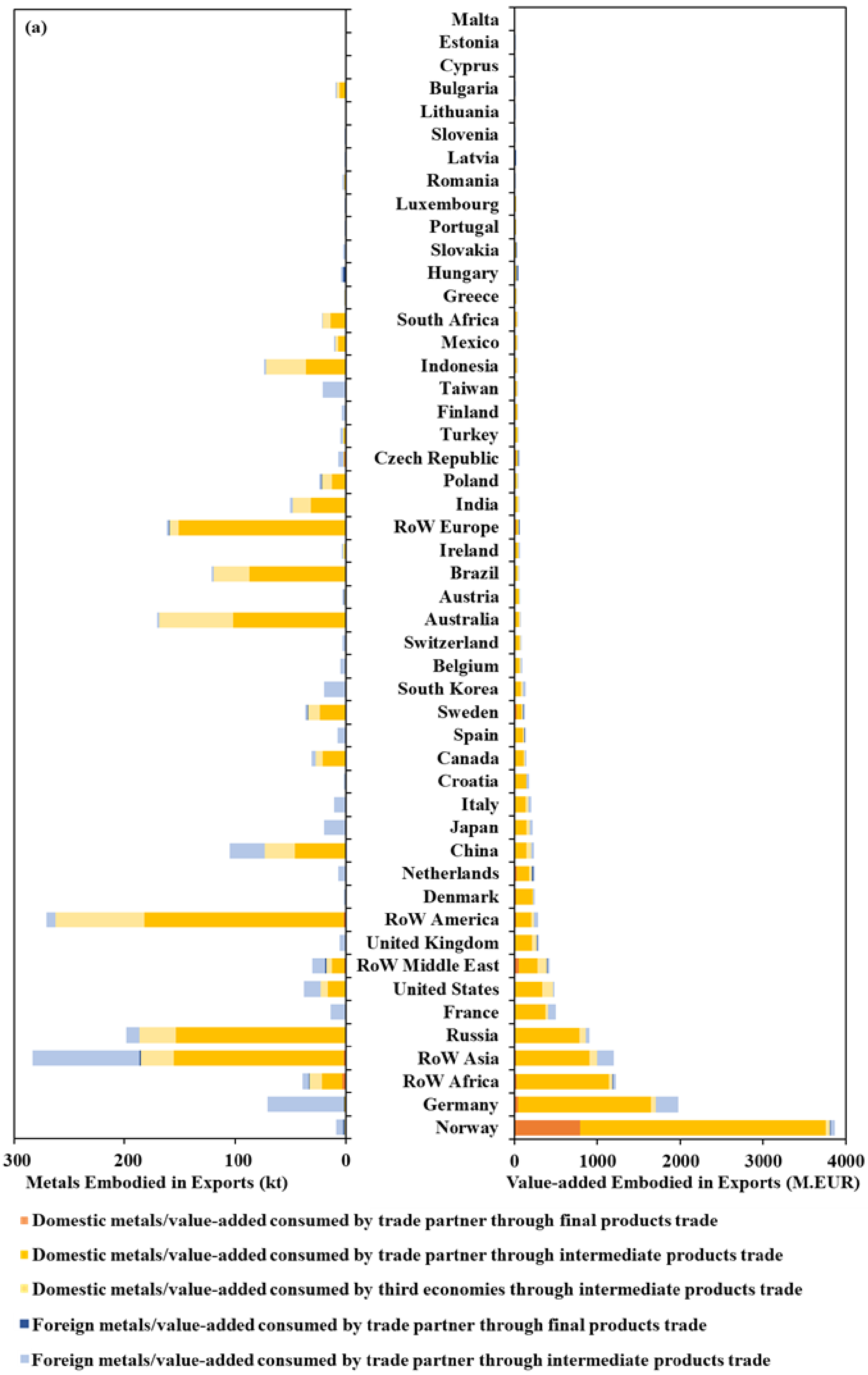
(a)
Malta
Estonia
Cyprus
Bulgaria
Lithuania
Slovenia
Latvia
Romania
Luxembourg
Portugal
Slovakia
Hungary
Greece
South Africa
Mexico
Indonesia
Taiwan
Finland
Turkey
Czech Republic
Poland
India
RoW Europe
Ireland
Brazil
Austria
Australia
Switzerland
Belgium
South Korea
Sweden
Spain
Canada
Croatia
Italy
Japan
China
Netherlands
Denmark
RoW America
United Kingdom
RoW Middle East
United States
France
Russia
RoW Asia
RoW Africa
Germany
Norway
300
200
100
0
Metals Embodied in Exports (kt)
0
1000
2000
3000
4000
Value-added Embodied in Exports (M.EUR)
Domestic metals/value-added consumed by trade partner through final products trade
Domestic metals/value-added consumed by trade partner through intermediate products trade
Domestic metals/value-added consumed by third economies through intermediate products trade
Foreign metals/value-added consumed by trade partner through final products trade
Foreign metals/value-added consumed by trade partner through intermediate products trade

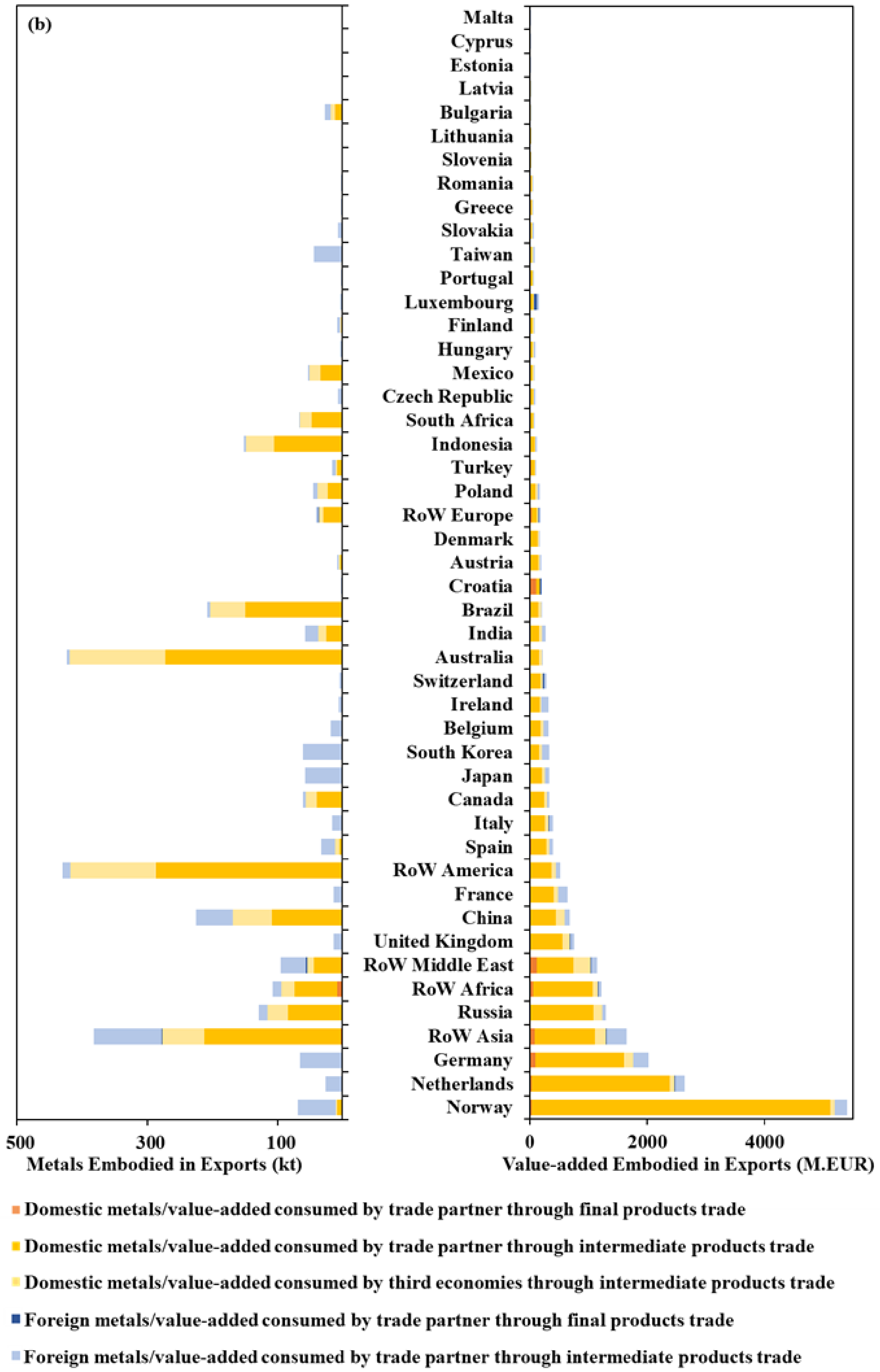

Supplementary Figure 7. The metals and value-added (million EUR, M.EUR) embodied in exports through global renewable power value chains for all the economies. (a) 2005, (b) 2015.

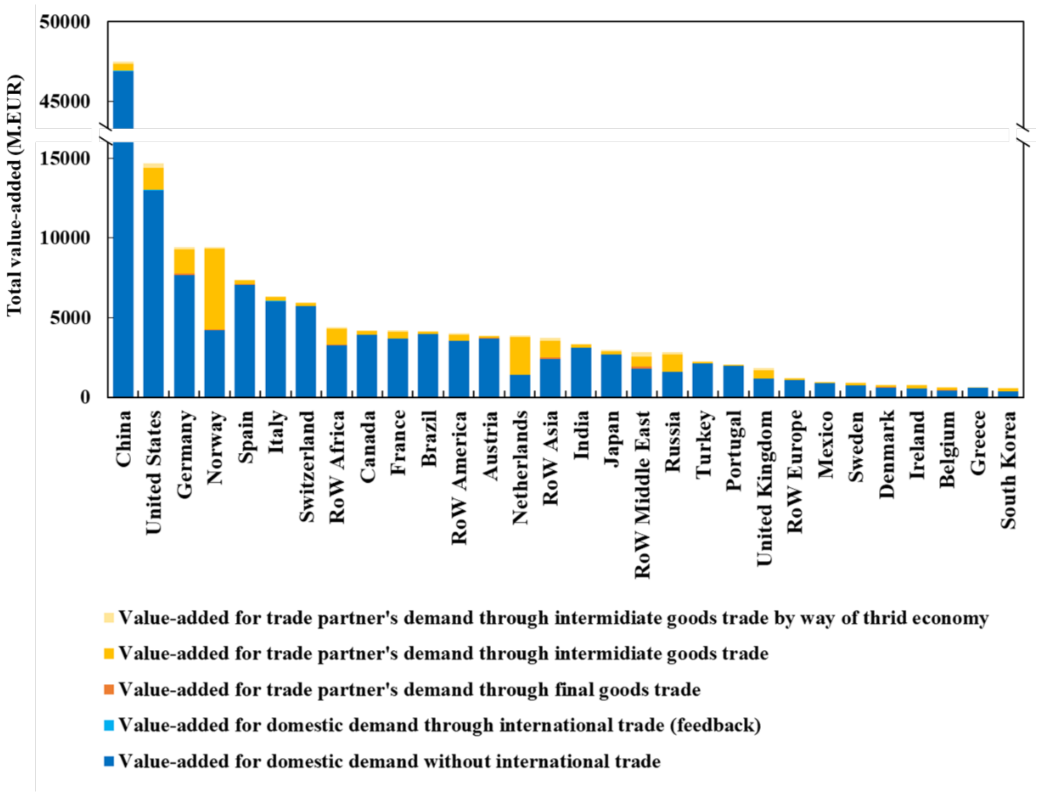

Supplementary Figure 8. Top 30 economies in terms of value added (million EUR, M.EUR) created by goods and services to meet both domestic and foreign renewable power demand. The blue bars represent value added created by goods and services to meet domestic renewable power demand, and the yellow bars represent that to meet foreign renewable power demand.

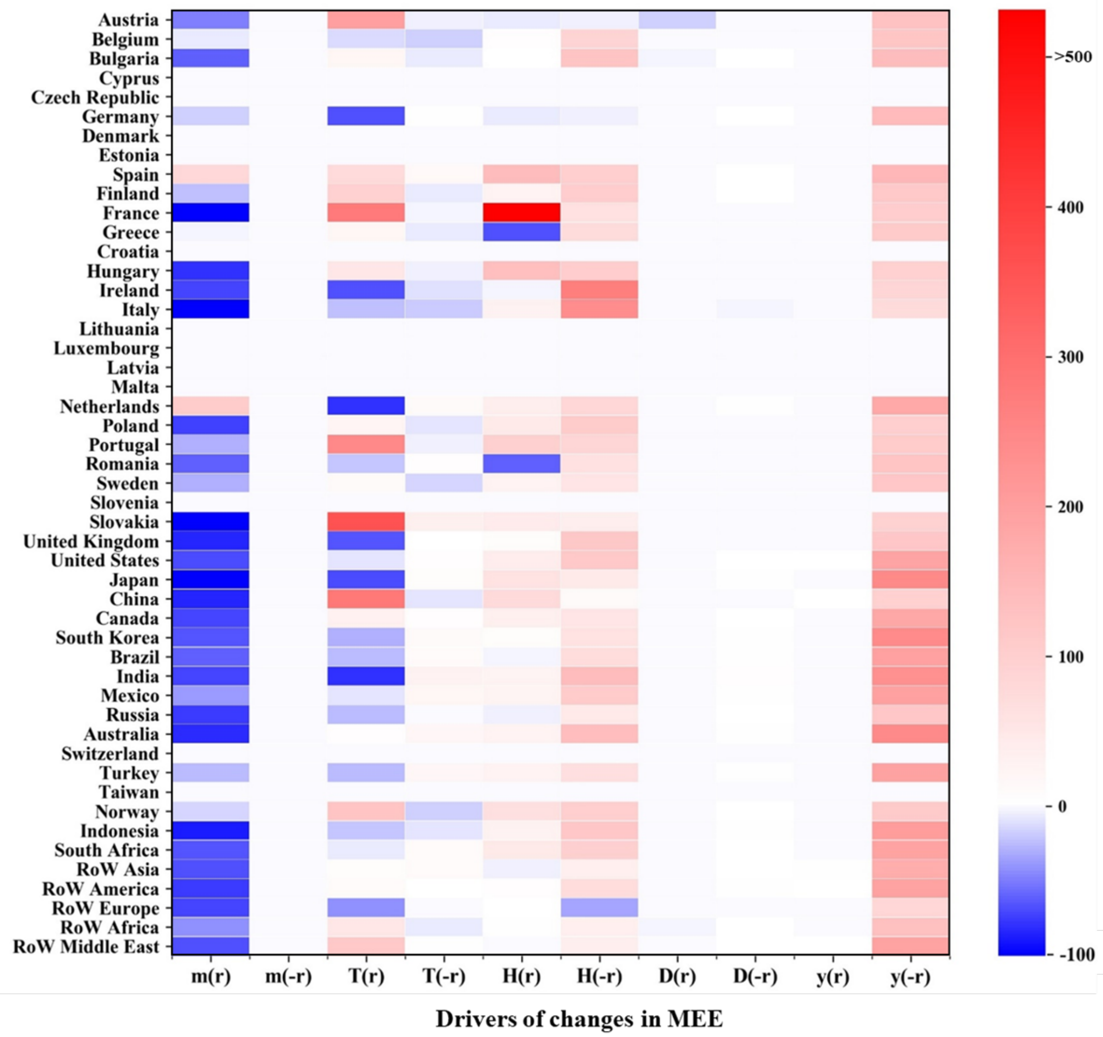

Supplementary Figure 9. The contribution of each factor to changes in metals embodied in export of all economies.

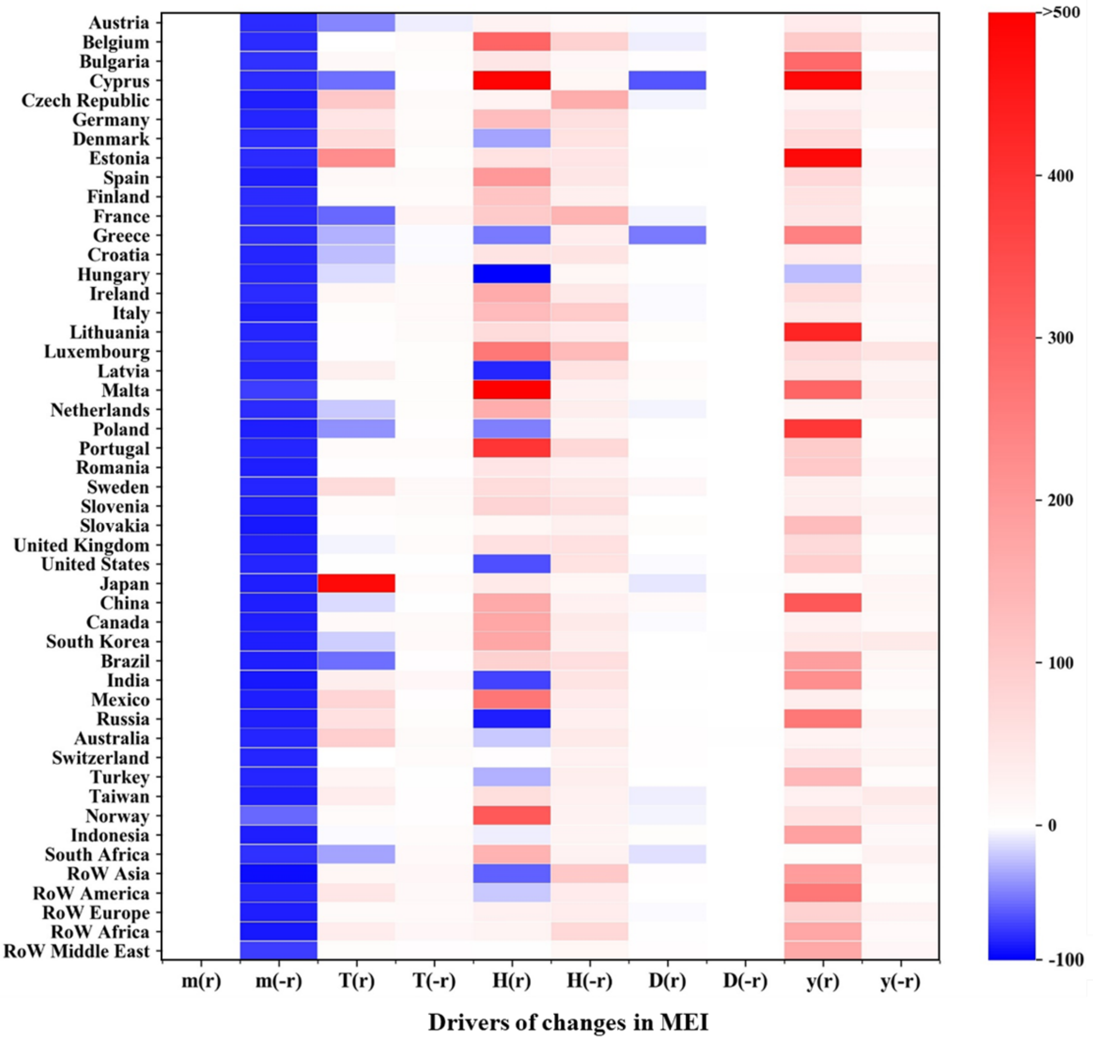

Supplementary Figure 10. The contribution of each factor to changes in metals embodied in import of all economies.

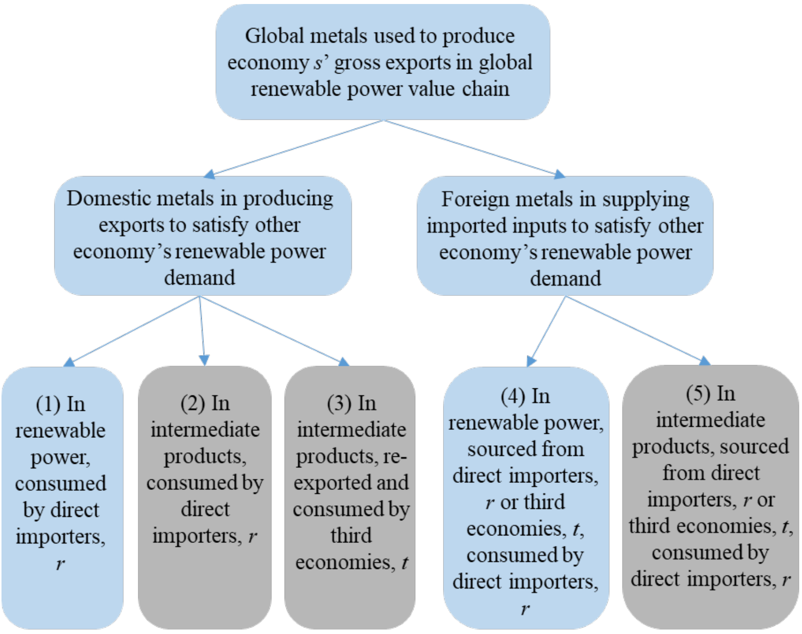

Supplementary Figure 11. Metal use embodied in an economy's gross exports by five global renewable power value chain routes.

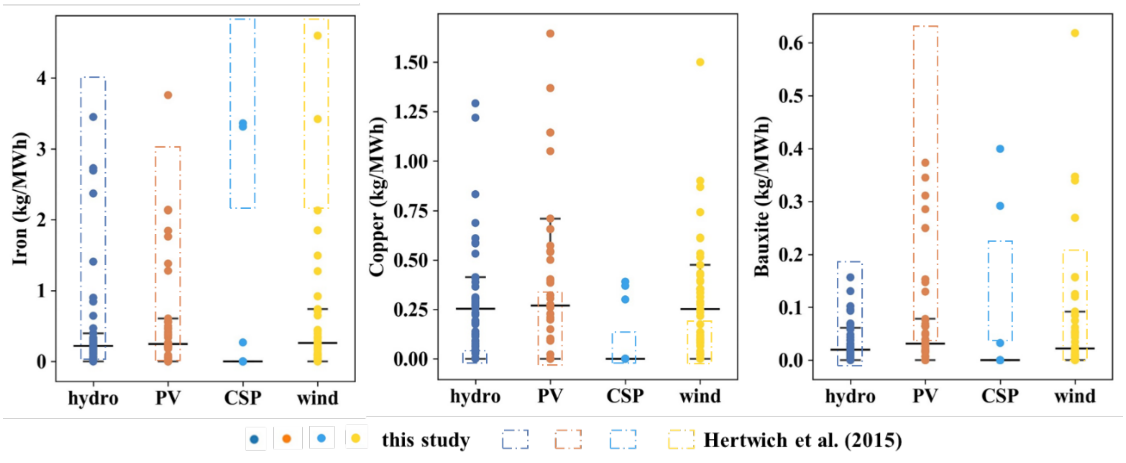

Supplementary Figure 12. The comparisons between our results with that of Hertwich et al. (2015).

Supplementary references

1. Leontief, W. Quantitative input and output relations in the economic system of the United States. *Rev. Econ. Stat*. **18**, 105–125 (1936).
2. Sébastien, M. & Ming, Y. Investigating Double Counting Terms in the Value-Added Decomposition of Gross Exports. *MPRA Pap.* 93877. Published 2020. Accessed May 03, 2023. https://mpra.ub.uni-muenchen.de/95437/1/MPRA_paper_95437.pdf.
3. Wang, Z., Wei, S. J. & Zhu, K. Quantifying International Production Sharing At the Bilateral and Sector Levels. *NBER Woking Pap.* **19667**, 1–127 (2013).
4. Koopman, R. Wang, Z. & Wei, S. Tracing value-added and double counting. *Am. Econ. Rev.* **104**, 459–494 (2014).
5. Xu, Y. & Dietzenbacher, E. A structural decomposition analysis of the emissions embodied in trade. *Ecol. Econ.* **101**, 10–20 (2014).
6. The United States. Geological Survey, 2015 Minerals Yearbook Austria. Published 2019. Accessed May 03, 2023. https://www.usgs.gov/media/files/mineral-industry-austria-2015-pdf.
7. The United States. Geological Survey, 2015 Minerals Yearbook, Indonesia. Published 2018. Accessed May 03, 2023. https://d9-wret.s3.us-west-2.amazonaws.com/assets/palladium/production/mineral-pubs/country/2015/myb3-2015-id.pdf.
8. British Geological Survey. World Mineral Production 2014-2018, Nottingham. Published 2020. Accessed May 03, 2023. https://www2.bgs.ac.uk/mineralsuk/download/world_statistics/2010s/WMP_2014_2018.pdf.
9. The International Renewable Energy Agency (IRENA), Renewable Energy Statistics 2015, Abu Dhabi. Published 2015. Accessed May 03, 2023. https://www.irena.org/Search?contentType=b28e6108-0ec4-4eb2-8d72-bdfb1e69c5c8&orderBy=Date.
10. Xu, Z. et al. Impacts of international trade on global sustainable development. Nat. Sustain. 3, 964–971 (2020).

11. Zhou, S. et al. China's power transformation may drastically change employment patterns in the power sector and its upstream supply chains. *Environ. Res. Lett.* **17**, 065005 (2022).
12. Wang, C. et al. Understanding the resource-use and environmental impacts of bioethanol production in China based on a MRIO-based hybrid LCA model. *Energy* **203**, 117877 (2020).
13. Hertwich, E. et al. Integrated life-cycle assessment of electricity-supply scenarios confirms global environmental benefit of low-carbon technologies. *Proc. Natl. Acad. Sci. USA* **112**, 6277-6282 (2014).
14. World Bank. World Bank Country and Lending Groups. Published 2018. Accessed June 06, 2023. https://datahelpdesk.worldbank.org/knowledgebase/articles/906519-world-bank-country-and-lending-groups..
